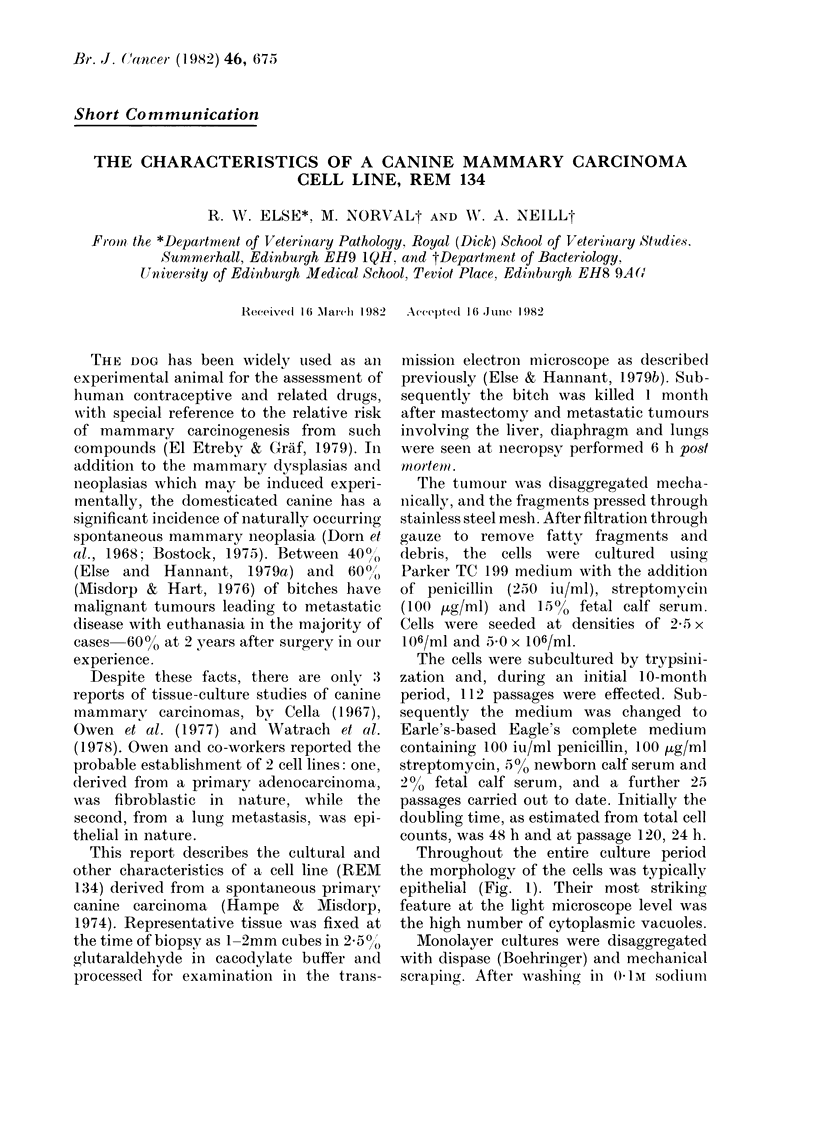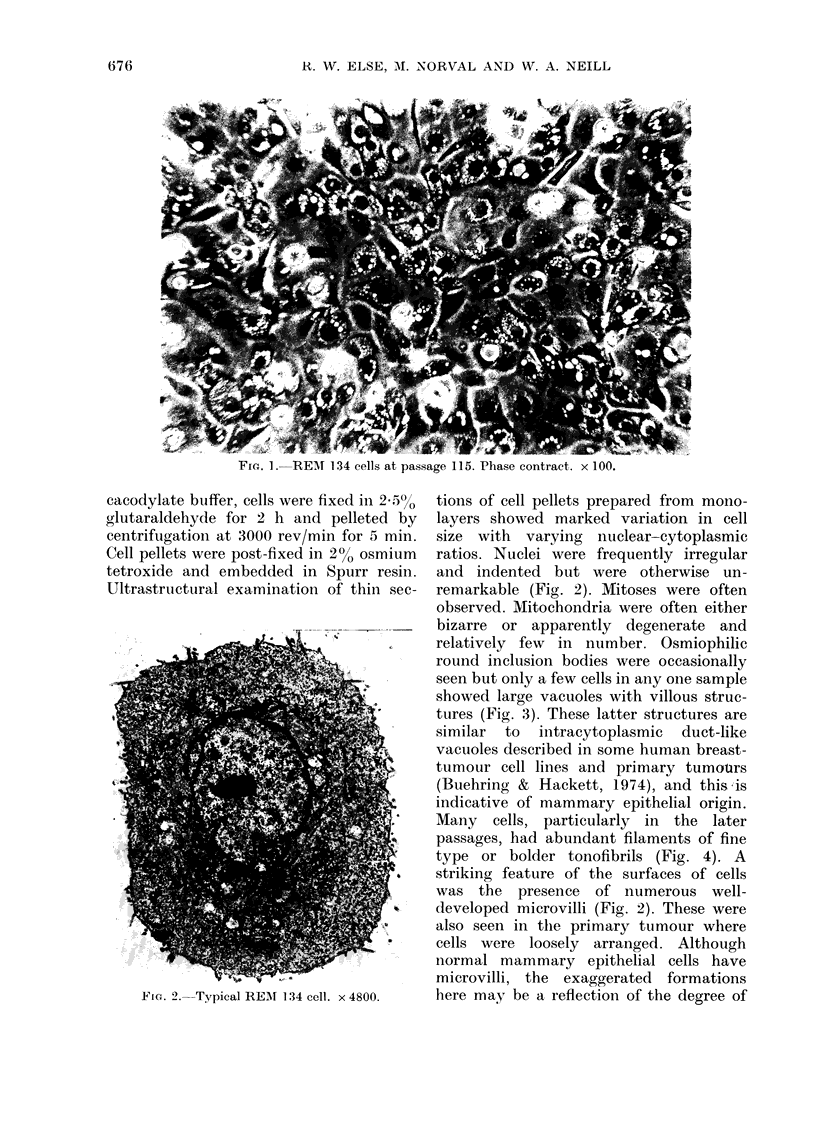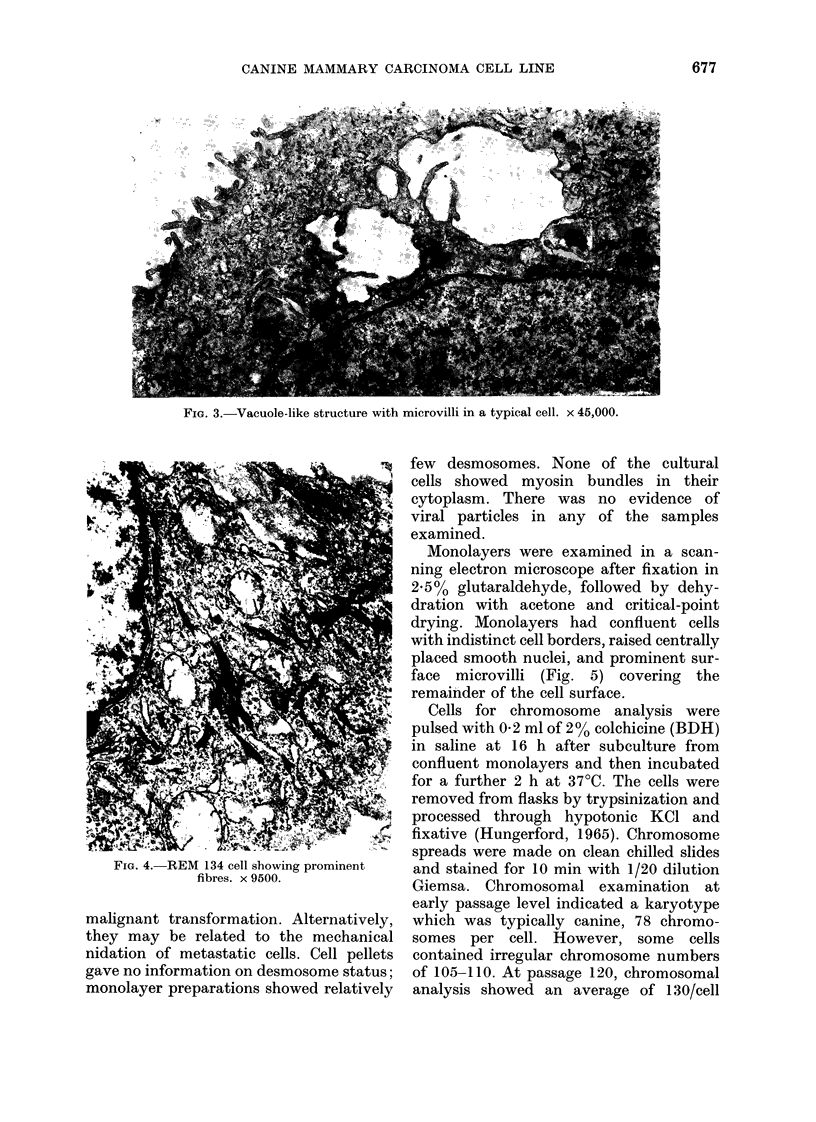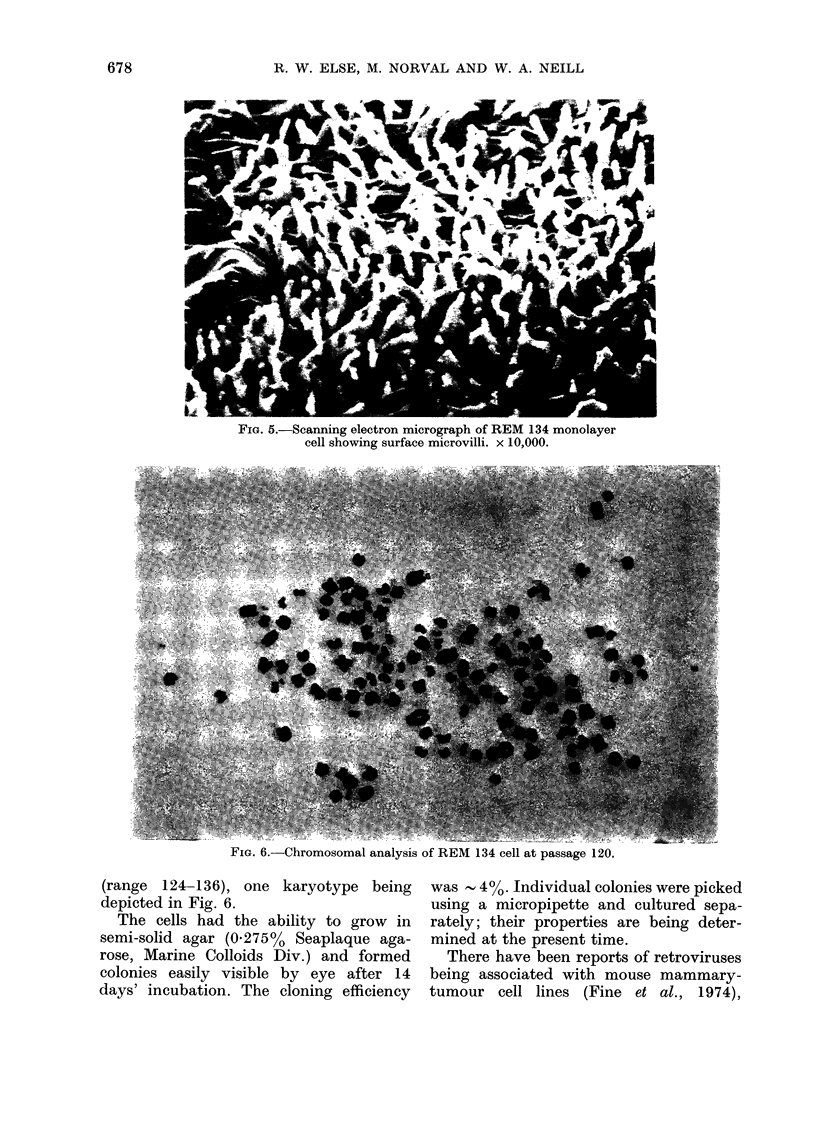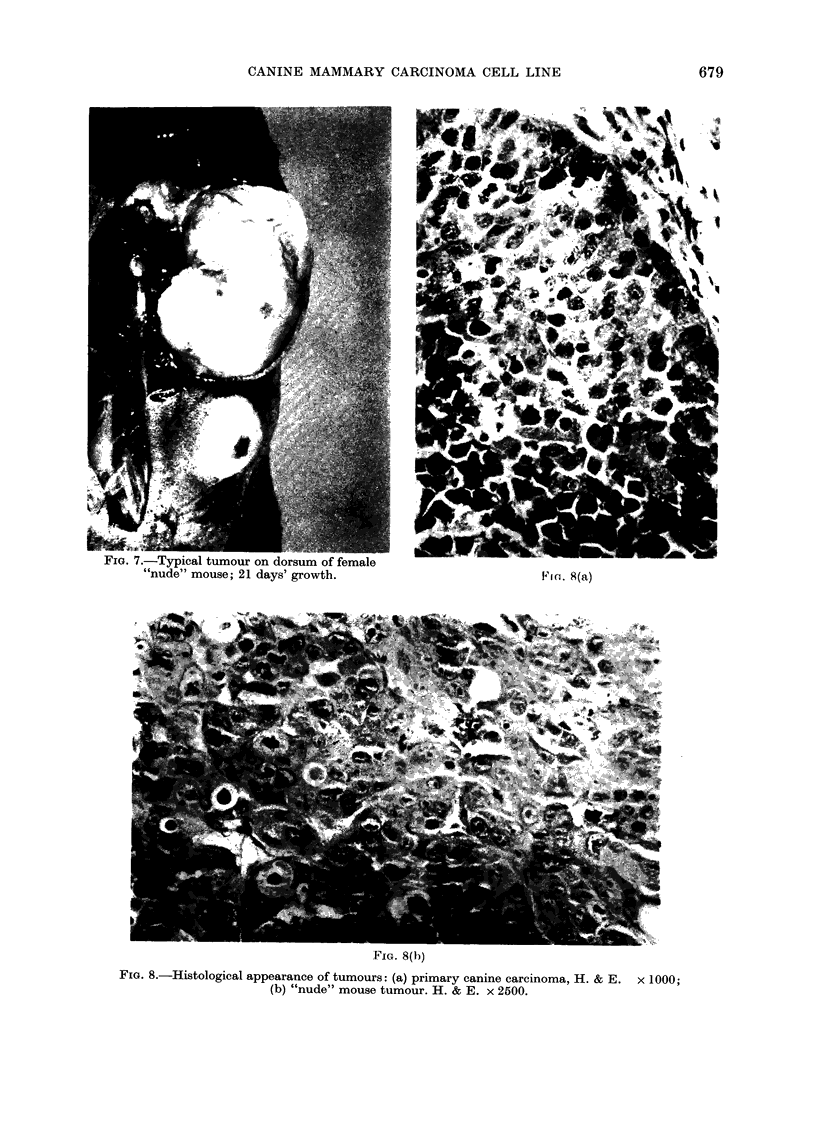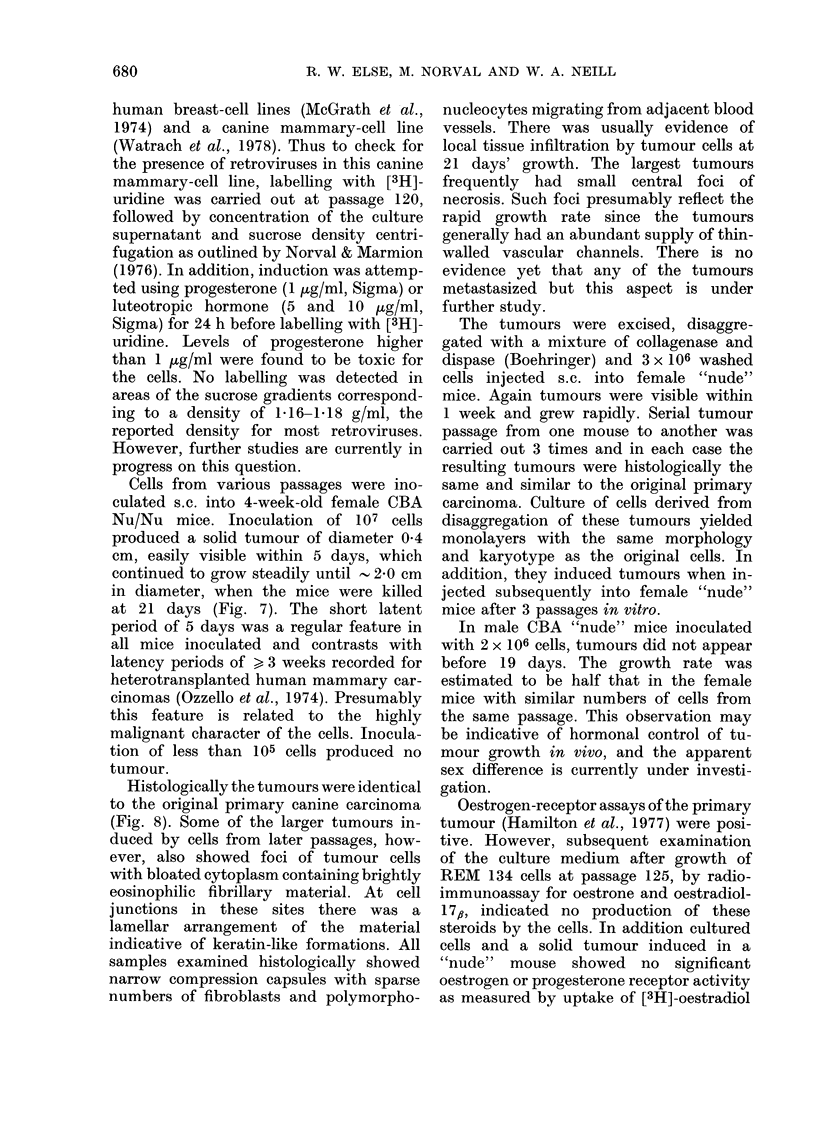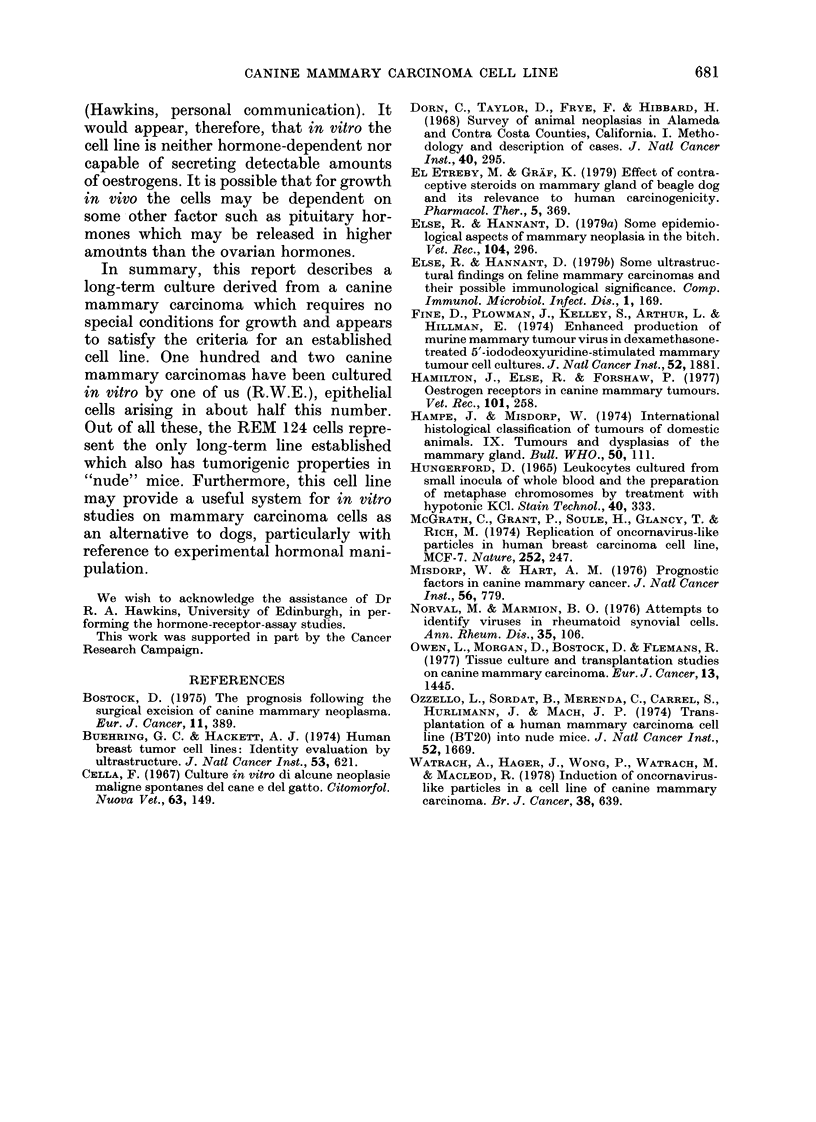# The characteristics of a canine mammary carcinoma cell line, REM 134.

**DOI:** 10.1038/bjc.1982.254

**Published:** 1982-10

**Authors:** R. W. Else, M. Norval, W. A. Neill

## Abstract

**Images:**


*Br. J. Cancer* (1982) **46**, 675

***Short Communication***

### THE CHARACTERISTICS OF A CANINE MAMMARY CARCINOMA CELL LINE, REM 134

R. W. ELSE*, M. NORVAL† AND W. A. NEILL†

*From the *Department of Veterinary Pathology, Royal (Dick) School of Veterinary Studies, Summerhall, Edinburgh EH9 1QH, and †Department of Bacteriology, University of Edinburgh Medical School, Teviot Place, Edinburgh EH8 9AG*

Received 16 March 1982 Accepted 16 June 1982

THE DOG has been widely used as an experimental animal for the assessment of human contraceptive and related drugs, with special reference to the relative risk of mammary carcinogenesis from such compounds (El Etreby & Gräf, 1979). In addition to the mammary dysplasias and neoplasias which may be induced experimentally, the domesticated canine has a significant incidence of naturally occurring spontaneous mammary neoplasia (Dorn *et al.*, 1968; Bostock, 1975). Between 40% (Else and Hannant, 1979*a*) and 60% (Misdorp & Hart, 1976) of bitches have malignant tumours leading to metastatic disease with euthanasia in the majority of cases—60% at 2 years after surgery in our experience.

Despite these facts, there are only 3 reports of tissue-culture studies of canine mammary carcinomas, by Cella (1967), Owen *et al.* (1977) and Watrach *et al.* (1978). Owen and co-workers reported the probable establishment of 2 cell lines: one, derived from a primary adenocarcinoma, was fibroblastic in nature, while the second, from a lung metastasis, was epithelial in nature.

This report describes the cultural and other characteristics of a cell line (REM 134) derived from a spontaneous primary canine carcinoma (Hampe & Misdorp, 1974). Representative tissue was fixed at the time of biopsy as 1–2mm cubes in 2·5% glutaraldehyde in cacodylate buffer and processed for examination in the transmission electron microscope as described previously (Else & Hannant, 1979*b*). Subsequently the bitch was killed 1 month after mastectomy and metastatic tumours involving the liver, diaphragm and lungs were seen at necropsy performed 6 h *post mortem*.

The tumour was disaggregated mechanically, and the fragments pressed through stainless steel mesh. After filtration through gauze to remove fatty fragments and debris, the cells were cultured using Parker TC 199 medium with the addition of penicillin (250 iu/ml), streptomycin (100 μg/ml) and 15% fetal calf serum. Cells were seeded at densities of $2{\cdot}5 \times 10^6$/ml and $5{\cdot}0 \times 10^6$/ml.

The cells were subcultured by trypsinization and, during an initial 10-month period, 112 passages were effected. Subsequently the medium was changed to Earle's-based Eagle's complete medium containing 100 iu/ml penicillin, 100 μg/ml streptomycin, 5% newborn calf serum and 2% fetal calf serum, and a further 25 passages carried out to date. Initially the doubling time, as estimated from total cell counts, was 48 h and at passage 120, 24 h.

Throughout the entire culture period the morphology of the cells was typically epithelial (Fig. 1). Their most striking feature at the light microscope level was the high number of cytoplasmic vacuoles.

Monolayer cultures were disaggregated with dispase (Boehringer) and mechanical scraping. After washing in 0·1M sodium

FIG. 1.—REM 134 cells at passage 115. Phase contract. ×100.

cacodylate buffer, cells were fixed in 2·5% glutaraldehyde for 2 h and pelleted by centrifugation at 3000 rev/min for 5 min. Cell pellets were post-fixed in 2% osmium tetroxide and embedded in Spurr resin. Ultrastructural examination of thin sections of cell pellets prepared from monolayers showed marked variation in cell size with varying nuclear–cytoplasmic ratios. Nuclei were frequently irregular and indented but were otherwise unremarkable (Fig. 2). Mitoses were often observed. Mitochondria were often either bizarre or apparently degenerate and relatively few in number. Osmiophilic round inclusion bodies were occasionally seen but only a few cells in any one sample showed large vacuoles with villous structures (Fig. 3). These latter structures are similar to intracytoplasmic duct-like vacuoles described in some human breast-tumour cell lines and primary tumours (Buehring & Hackett, 1974), and this is indicative of mammary epithelial origin. Many cells, particularly in the later passages, had abundant filaments of fine type or bolder tonofibrils (Fig. 4). A striking feature of the surfaces of cells was the presence of numerous well-developed microvilli (Fig. 2). These were also seen in the primary tumour where cells were loosely arranged. Although normal mammary epithelial cells have microvilli, the exaggerated formations here may be a reflection of the degree of

FIG. 2.—Typical REM 134 cell. ×4800.

FIG. 3.—Vacuole-like structure with microvilli in a typical cell. × 45,000.

FIG. 4.—REM 134 cell showing prominent fibres. × 9500.

malignant transformation. Alternatively, they may be related to the mechanical nidation of metastatic cells. Cell pellets gave no information on desmosome status; monolayer preparations showed relatively few desmosomes. None of the cultural cells showed myosin bundles in their cytoplasm. There was no evidence of viral particles in any of the samples examined.

Monolayers were examined in a scanning electron microscope after fixation in 2·5% glutaraldehyde, followed by dehydration with acetone and critical-point drying. Monolayers had confluent cells with indistinct cell borders, raised centrally placed smooth nuclei, and prominent surface microvilli (Fig. 5) covering the remainder of the cell surface.

Cells for chromosome analysis were pulsed with 0·2 ml of 2% colchicine (BDH) in saline at 16 h after subculture from confluent monolayers and then incubated for a further 2 h at 37°C. The cells were removed from flasks by trypsinization and processed through hypotonic KCl and fixative (Hungerford, 1965). Chromosome spreads were made on clean chilled slides and stained for 10 min with 1/20 dilution Giemsa. Chromosomal examination at early passage level indicated a karyotype which was typically canine, 78 chromosomes per cell. However, some cells contained irregular chromosome numbers of 105–110. At passage 120, chromosomal analysis showed an average of 130/cell

FIG. 5.—Scanning electron micrograph of REM 134 monolayer cell showing surface microvilli. ×10,000.

FIG. 6.—Chromosomal analysis of REM 134 cell at passage 120.

(range 124–136), one karyotype being depicted in Fig. 6.

The cells had the ability to grow in semi-solid agar (0·275% Seaplaque agarose, Marine Colloids Div.) and formed colonies easily visible by eye after 14 days' incubation. The cloning efficiency was ~4%. Individual colonies were picked using a micropipette and cultured separately; their properties are being determined at the present time.

There have been reports of retroviruses being associated with mouse mammary-tumour cell lines (Fine *et al.*, 1974),

FIG. 7.—Typical tumour on dorsum of female "nude" mouse; 21 days' growth.

FIG. 8(a)

FIG. 8(b)

FIG. 8.—Histological appearance of tumours: (a) primary canine carcinoma, H. & E. ×1000; (b) "nude" mouse tumour. H. & E. ×2500.

human breast-cell lines (McGrath *et al.*, 1974) and a canine mammary-cell line (Watrach *et al.*, 1978). Thus to check for the presence of retroviruses in this canine mammary-cell line, labelling with [$^3$H]-uridine was carried out at passage 120, followed by concentration of the culture supernatant and sucrose density centrifugation as outlined by Norval & Marmion (1976). In addition, induction was attempted using progesterone (1 $\mu$g/ml, Sigma) or luteotropic hormone (5 and 10 $\mu$g/ml, Sigma) for 24 h before labelling with [$^3$H]-uridine. Levels of progesterone higher than 1 $\mu$g/ml were found to be toxic for the cells. No labelling was detected in areas of the sucrose gradients corresponding to a density of 1·16–1·18 g/ml, the reported density for most retroviruses. However, further studies are currently in progress on this question.

Cells from various passages were inoculated s.c. into 4-week-old female CBA Nu/Nu mice. Inoculation of $10^7$ cells produced a solid tumour of diameter 0·4 cm, easily visible within 5 days, which continued to grow steadily until $\sim$2·0 cm in diameter, when the mice were killed at 21 days (Fig. 7). The short latent period of 5 days was a regular feature in all mice inoculated and contrasts with latency periods of $\geqslant$3 weeks recorded for heterotransplanted human mammary carcinomas (Ozzello *et al.*, 1974). Presumably this feature is related to the highly malignant character of the cells. Inoculation of less than $10^5$ cells produced no tumour.

Histologically the tumours were identical to the original primary canine carcinoma (Fig. 8). Some of the larger tumours induced by cells from later passages, however, also showed foci of tumour cells with bloated cytoplasm containing brightly eosinophilic fibrillary material. At cell junctions in these sites there was a lamellar arrangement of the material indicative of keratin-like formations. All samples examined histologically showed narrow compression capsules with sparse numbers of fibroblasts and polymorphonucleocytes migrating from adjacent blood vessels. There was usually evidence of local tissue infiltration by tumour cells at 21 days' growth. The largest tumours frequently had small central foci of necrosis. Such foci presumably reflect the rapid growth rate since the tumours generally had an abundant supply of thin-walled vascular channels. There is no evidence yet that any of the tumours metastasized but this aspect is under further study.

The tumours were excised, disaggregated with a mixture of collagenase and dispase (Boehringer) and $3 \times 10^6$ washed cells injected s.c. into female "nude" mice. Again tumours were visible within 1 week and grew rapidly. Serial tumour passage from one mouse to another was carried out 3 times and in each case the resulting tumours were histologically the same and similar to the original primary carcinoma. Culture of cells derived from disaggregation of these tumours yielded monolayers with the same morphology and karyotype as the original cells. In addition, they induced tumours when injected subsequently into female "nude" mice after 3 passages *in vitro*.

In male CBA "nude" mice inoculated with $2 \times 10^6$ cells, tumours did not appear before 19 days. The growth rate was estimated to be half that in the female mice with similar numbers of cells from the same passage. This observation may be indicative of hormonal control of tumour growth *in vivo*, and the apparent sex difference is currently under investigation.

Oestrogen-receptor assays of the primary tumour (Hamilton *et al.*, 1977) were positive. However, subsequent examination of the culture medium after growth of REM 134 cells at passage 125, by radioimmunoassay for oestrone and oestradiol-$17_\beta$, indicated no production of these steroids by the cells. In addition cultured cells and a solid tumour induced in a "nude" mouse showed no significant oestrogen or progesterone receptor activity as measured by uptake of [$^3$H]-oestradiol

(Hawkins, personal communication). It would appear, therefore, that *in vitro* the cell line is neither hormone-dependent nor capable of secreting detectable amounts of oestrogens. It is possible that for growth *in vivo* the cells may be dependent on some other factor such as pituitary hormones which may be released in higher amounts than the ovarian hormones.

In summary, this report describes a long-term culture derived from a canine mammary carcinoma which requires no special conditions for growth and appears to satisfy the criteria for an established cell line. One hundred and two canine mammary carcinomas have been cultured *in vitro* by one of us (R.W.E.), epithelial cells arising in about half this number. Out of all these, the REM 124 cells represent the only long-term line established which also has tumorigenic properties in "nude" mice. Furthermore, this cell line may provide a useful system for *in vitro* studies on mammary carcinoma cells as an alternative to dogs, particularly with reference to experimental hormonal manipulation.

We wish to acknowledge the assistance of Dr R. A. Hawkins, University of Edinburgh, in performing the hormone-receptor-assay studies.

This work was supported in part by the Cancer Research Campaign.

### REFERENCES

BOSTOCK, D. (1975) The prognosis following the surgical excision of canine mammary neoplasma. *Eur. J. Cancer*, **11**, 389.

BUEHRING, G. C. & HACKETT, A. J. (1974) Human breast tumor cell lines: Identity evaluation by ultrastructure. *J. Natl Cancer Inst.*, **53**, 621.

CELLA, F. (1967) Culture *in vitro* di alcune neoplasie maligne spontanes del cane e del gatto. *Citomorfol. Nuova Vet.*, **63**, 149.

DORN, C., TAYLOR, D., FRYE, F. & HIBBARD, H. (1968) Survey of animal neoplasias in Alameda and Contra Costa Counties, California. I. Methodology and description of cases. *J. Natl Cancer Inst.*, **40**, 295.

EL ETREBY, M. & GRÄF, K. (1979) Effect of contraceptive steroids on mammary gland of beagle dog and its relevance to human carcinogenicity. *Pharmacol. Ther.*, **5**, 369.

ELSE, R. & HANNANT, D. (1979*a*) Some epidemiological aspects of mammary neoplasia in the bitch. *Vet. Rec.*, **104**, 296.

ELSE, R. & HANNANT, D. (1979*b*) Some ultrastructural findings on feline mammary carcinomas and their possible immunological significance. *Comp. Immunol. Microbiol. Infect. Dis.*, **1**, 169.

FINE, D., PLOWMAN, J., KELLEY, S., ARTHUR, L. & HILLMAN, E. (1974) Enhanced production of murine mammary tumour virus in dexamethasone-treated 5′-iododeoxyuridine-stimulated mammary tumour cell cultures. *J. Natl Cancer Inst.*, **52**, 1881.

HAMILTON, J., ELSE, R. & FORSHAW, P. (1977) Oestrogen receptors in canine mammary tumours. *Vet. Rec.*, **101**, 258.

HAMPE, J. & MISDORP, W. (1974) International histological classification of tumours of domestic animals. IX. Tumours and dysplasias of the mammary gland. *Bull. WHO.*, **50**, 111.

HUNGERFORD, D. (1965) Leukocytes cultured from small inocula of whole blood and the preparation of metaphase chromosomes by treatment with hypotonic KCl. *Stain Technol.*, **40**, 333.

MCGRATH, C., GRANT, P., SOULE, H., GLANCY, T. & RICH, M. (1974) Replication of oncornavirus-like particles in human breast carcinoma cell line, MCF-7. *Nature*, **252**, 247.

MISDORP, W. & HART, A. M. (1976) Prognostic factors in canine mammary cancer. *J. Natl Cancer Inst.*, **56**, 779.

NORVAL, M. & MARMION, B. O. (1976) Attempts to identify viruses in rheumatoid synovial cells. *Ann. Rheum. Dis.*, **35**, 106.

OWEN, L., MORGAN, D., BOSTOCK, D. & FLEMANS, R. (1977) Tissue culture and transplantation studies on canine mammary carcinoma. *Eur. J. Cancer*, **13**, 1445.

OZZELLO, L., SORDAT, B., MERENDA, C., CARREL, S., HURLIMANN, J. & MACH, J. P. (1974) Transplantation of a human mammary carcinoma cell line (BT20) into nude mice. *J. Natl Cancer Inst.*, **52**, 1669.

WATRACH, A., HAGER, J., WONG, P., WATRACH, M. & MACLEOD, R. (1978) Induction of oncornavirus-like particles in a cell line of canine mammary carcinoma. *Br. J. Cancer*, **38**, 639.